# Necrotizing soft tissue infection of the abdominal wall following vaginal delivery in a ruptured uterus: a case series from Nigeria

**DOI:** 10.1093/jscr/rjaf675

**Published:** 2025-09-28

**Authors:** Samuel Onuh, Aurelie Godard, Carlos Pilasi, Djamila A Salifou, Fatima Aliyu, Mariam Nakisembo, Abdulwahab H M Mohamed, Yusuf Ali, Hamisu Yakubu, Katharina Weizsacker

**Affiliations:** Obstetrics and Gynecology, Medecins Sans Frontieres (OCP), 3JJG+5P9, Kafin Hausa Road, Jahun 720103, Nigeria; Obstetrics and Gynecology, Medecins Sans Frontieres, 14-34 Avenue Jean Jaurès, Paris 75019, France; Surgery, Medecins Sans Frontieres, 14-34 Avenue Jean Jaurès, Paris 75019, France; Obstetrics and Gynecology, Medecins Sans Frontieres, 14-34 Avenue Jean Jaurès, Paris 75019, France; Obstetrics and Gynecology, Medecins Sans Frontieres (OCP), 3JJG+5P9, Kafin Hausa Road, Jahun 720103, Nigeria; Obstetrics and Gynecology, Medecins Sans Frontieres, 14-34 Avenue Jean Jaurès, Paris 75019, France; Obstetrics and Gynecology, Medecins Sans Frontieres (OCP), 3JJG+5P9, Kafin Hausa Road, Jahun 720103, Nigeria; Anesthesia, Ahmadu Bello University Teaching Hospital, A126, Ungwan Doka, Zaria 810105, Nigeria; Anesthesia, Ahmadu Bello University Teaching Hospital, A126, Ungwan Doka, Zaria 810105, Nigeria; Obstetrics and Gynecology, Medecins Sans Frontieres, 14-34 Avenue Jean Jaurès, Paris 75019, France

**Keywords:** necrotizing fasciitis, obstetric surgery, Nigeria

## Abstract

Necrotizing soft tissue infections (NSTIs) in pregnancy and puerperium are rare but life-threatening, with high morbidity and mortality. Diagnosis is often delayed due to nonspecific symptoms. While commonly linked to cesarean delivery and pelvic surgeries, NSTIs can also arise in spontaneous vaginal deliveries. We report two postpartum NSTI cases in women following vaginal delivery in a silent uterine rupture that were managed in a low-resource setting in a rural part of Nigeria. Puerperal NSTIs require early recognition and aggressive management. Uterine rupture may be an unrecognized source of infection. Timely surgical debridement, broad-spectrum antibiotics, and supportive care are critical for survival. Diagnosis and management can be challenging in low-resource settings, and clinical suspicion with early surgical and medical intervention is essential for a positive outcome.

## Introduction

Necrotizing soft tissue infections (NSTIs) are a rare but life-threatening condition in obstetrics, with high morbidity and mortality [1]. Data on epidemiology, clinical features, and outcomes remain limited [2]. It can progress rapidly, causing necrosis of muscle, fascia, and surrounding tissues [3]. General incidence is 0.86 per 100 000 per year [4] and published mortality rates vary (8%–80%) [5]. However, little is known about NSTI in pregnancy and puerperium, partially because of inconsistent terminology [6]. The absence of pathognomonic signs makes early diagnosis difficult, leading to delayed intervention and poor prognoses [5]. Mortality in obstetric patients is 10%–40% [7–9]. NSTI in obstetrical patients often follows surgery (cesarean delivery, hysterectomy, other pelvic/abdominal procedures) or septic abortion, especially in women with obesity, hypertension, or diabetes [10–13].

NSTI can cause sepsis, organ failure, and death. It typically affects the extremities, perineum, and/or abdominal wall [7, 8]. In obstetrics, NSTI most commonly involves the breast, perineum, and cesarean wound, and infections are often polymicrobial.

NSTI requires urgent intervention. Treatment includes surgical debridement, broad-spectrum antibiotics (empirical treatment should be started early and include anaerobic bacteria), and supportive care. Surgical debridement is essential for infection control and may require multiple procedures. Hyperbaric oxygen therapy may help tissue healing but remains controversial [14–17] and is not available in many settings.

Jigawa State is a rural area in northern Nigeria with a high maternal mortality rate (1012 maternal deaths per 100 000 live births) [18], largely due to limited access to quality healthcare, especially for pregnant women. Economic, cultural, and logistical barriers make it hard for most women to access skilled birth attendance or emergency obstetric care. We present two cases with no prior surgery, trauma, or other known risk factors, managed successfully at the Medecins Sans Frontieres hospital in Jahun, Jigawa State.

## Case one

**Figure 1:**
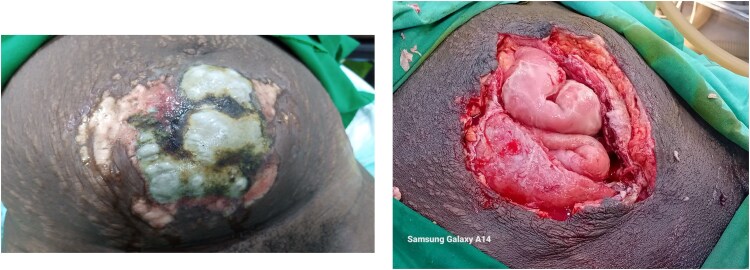
Case 1: Wound at presentation and after 4 days of continuous debridement.

A 30-year-old P10 + 0 7A presented 10 days after normal vaginal delivery (in a primary health care center) of a life term infant. The patient complained of abdominal pain and a lower abdominal rash with purulent discharge. She was alert, afebrile (36.5°C), tachycardic (131/min), and anemic (Hb 7.8 g/dl). Examination revealed generalized abdominal tenderness, guarding, foul-smelling lochiae, and a large necrotic sub-umbilical area (Fig. 1). Ultrasound suggested some retained placental tissue but was otherwise inconclusive.

Laparotomy showed extensive necrosis, involving skin, subcutaneous tissue, adipose tissue, and rectus sheath. There was a right uterine rupture with cervical laceration. Hysterectomy and debridement left a large anterior abdominal wall defect, closed with a Bogotá bag. Multiple wound debridements followed by staged closure were performed with the remaining wound healing by secondary intention (Fig. 3). She received broad-spectrum antibiotics (including amoxicillin/clavulanic acid, clindamycin, and metronidazole), analgesics, and supportive care, recovering well and being discharged after 4 weeks.

## Case two

A 30-year-old P5 presented 5 days after home delivery of a life term infant with fever, offensive vaginal discharge, lower abdominal pain, and superficial blistering with a wound on the anterior abdominal wall without a history of trauma.

**Figure 2:**
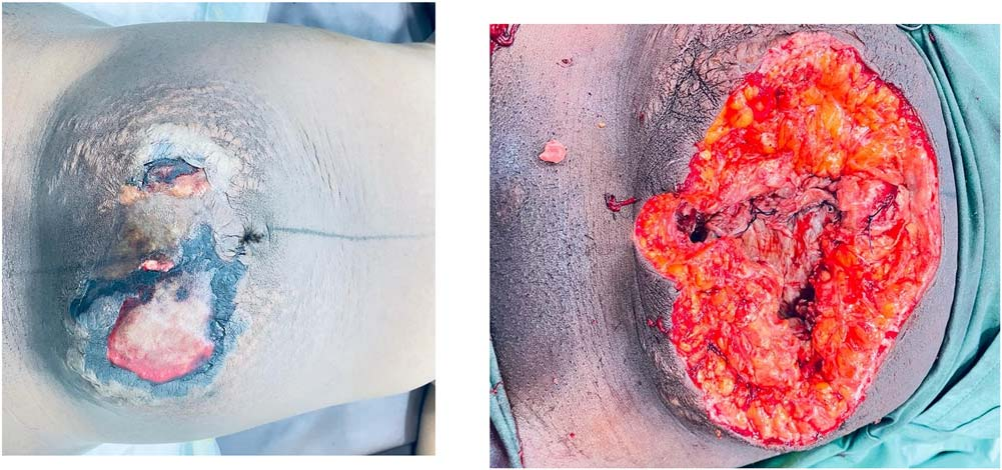
Case 2: Anterior abdominal wall wound at presentation and following 3 days of continuous debridement.

On admission, she was febrile (38.1°C), tachycardic (130/min), and mildly anemic (Hb 9 g/dl). Examination revealed two edematous areas (~10 × 10 cm) with foul-smelling discharge but no rigidity or guarding (Fig. 2). Ultrasound showed no free fluid.

Laparotomy revealed a large necrotic area involving the skin, subcutaneous tissue, rectus muscles and sheath, along with a left lower segment uterine rupture, and hysterectomy was done. She underwent multiple wound debridements.

On postoperative day seven, she developed acute vaginal and intra-abdominal bleeding, requiring left uterine artery ligation and further intra-abdominal debridement of necrotic tissue. She received broad-spectrum antibiotics (amoxicillin/clavulanic acid, clindamycin, metronidazole). The skin was subsequently reapproximated. The remainder of the recovery was uncomplicated, and she was doing well at follow-up.

**Figure 3:**
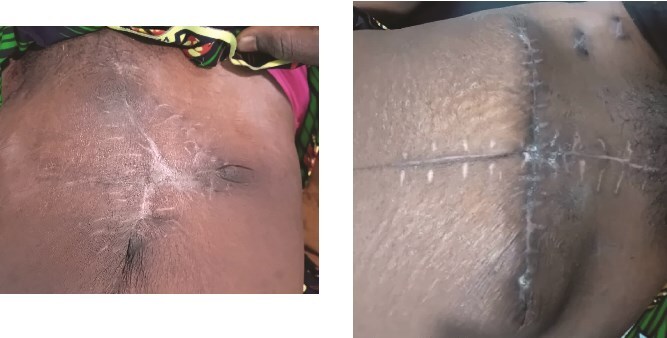
Case 1 (left) and 2 (right): Anterior abdominal walls of both patients 6 weeks after surgery.

Both patients underwent subtotal hysterectomy with serial wound debridements, consistent with the standard of aggressive surgical debridement. Although extensive abdominal wall debridement often necessitates split skin grafting, serial wound approximation allowed closure without grafts, aided by the laxity of skin and abdominal wall tissues in the postpartum period. This has been described previously in the literature [15, 17, 19]. Gradual abdominoplasty with initial closure of the rectus sheath and muscle apposition to protect the bowel was done, followed by stepwise skin closure over 2–3 days using tension sutures while protecting the skin by passing the suture through cut IV tubing. No tissue or skin grafting was required, and healing was complete in both women after 4–6 weeks (Fig. 3).

Additional care included high-dose antibiotics (amoxicillin-clavulanic acid, clindamycin, metronidazole), nutritional support, blood transfusion, and fluid management, following established NSTI treatment protocols [15–17].

## Discussion

Abdominal wall NSTI following vaginal delivery is exceedingly rare. The underlying cause in our two cases was uterine rupture in what was initially assumed to be uncomplicated deliveries in multiparous patients.

Pregnancy, and the puerperium period, can mask early NSTI signs—fever, pain, erythema—and complicate diagnosis [9, 20]. However, both of our patients presented with marked skin changes (Figs 1 and 2), raising the clinical suspicion of NSTI. These changes were more prominent on the side of the uterine rupture, supporting a continuous spread of the infection.

NSTIs can originate from the uterus without external incisions. Most reports involve cesarean or perineal trauma. But NSTI with uterine necrosis requiring hysterectomy [21], and retroperitoneal iliopsoas NSTI after vaginal delivery [22] have been described.

Our report shows that in cases of postpartum NSTI, all potential sources of trauma—including uterine rupture—must be considered, even if the delivery was apparently uncomplicated.

Rapid diagnosis can be challenging in settings without access to imaging studies such as computed tomography or comprehensive laboratory support. Nevertheless, we show that these cases can be managed well in a low-resource setting if there is clinical awareness, appropriate management with meticulous surgical debridement to excise necrotic tissue, and comprehensive supportive care that is promptly initiated. This includes empirical antibiotic therapy, even when access to microbiological analyses is absent, as well as fluid management and nutritional support.

Reports of NSTI involving the abdominal wall after spontaneous vaginal delivery are virtually nonexistent in the literature, with most documented cases occurring post-cesarean section. This case series contributes to the limited body of knowledge of this rare but life-threatening complication.
